# Minimum Contradiction Matrices in Whole Genome Phylogenies

**DOI:** 10.4137/ebo.s909

**Published:** 2008-07-21

**Authors:** Marc Thuillard

**Affiliations:** Belimo Automation AG, CH-8340 Hinwil, Switzerland

**Keywords:** phylogenetic trees, whole genome phylogeny, minimum contradiction, split network

## Abstract

Minimum contradiction matrices are a useful complement to distance-based phylogenies. A minimum contradiction matrix represents phylogenetic information under the form of an ordered distance matrix *Y_i_*_,_ *_j_^n^*. A matrix element corresponds to the distance from a reference vertex *n* to the path (*i*, *j*). For an X-tree or a split network, the minimum contradiction matrix is a Robinson matrix. It therefore fulfills all the inequalities defining perfect order: *Y_i_*_,_ *_j_^n^* ≥ *Y_i_*_,_*_k_^n^*_,_ *Y_k j_^n^* ≥ *Y_k_*_,_ *_I_^n^*, *i* ≤ j ≤ *k* < *n*. In real phylogenetic data, some taxa may contradict the inequalities for perfect order. Contradictions to perfect order correspond to deviations from a tree or from a split network topology. Efficient algorithms that search for the best order are presented and tested on whole genome phylogenies with 184 taxa including many Bacteria, Archaea and Eukaryota. After optimization, taxa are classified in their correct domain and phyla. Several significant deviations from perfect order correspond to well-documented evolutionary events.

## 1. Introduction

The discovery of the importance of lateral transfers, losses and duplications events in the evolution of genetic sequences has motivated the development of new approaches to graphically represent phylogenies. Methods like NeighborNet (Bryant and Moulton, 2004), T-Rex (Makarenkov et al. 2006), SplitTrees (Bandelt and Dress, 1992; Dress and Huson, 2004; Huson, 1998), Qnet (Grünewald et al. 2006), Pyramids (Bertrand and Diday, 1985), Tree of Life (Kunin et al. 2005a) allow visualizing deviations from a tree topology. All these methods have in common that they summarize the information in the form of a planar network. Deviations from an X-tree are often represented by supplementary edges (Makarenkov et al. 2006; Nakhleh et al. 2004) that create cycles in the graph.

Phylogenetic information can be represented by a distance matrix *Y_i_*_,_ *_j_^n^*. For an X-tree, the elements of the distance matrix *Y_i_*_,_ *_j_^n^* correspond to the distance from a reference taxon *n* to the path (*i*, *j*). The taxa can be ordered through permutations, so that the distance matrix is a Robinson matrix (Bertrand and Diday, 1985), with values of both rows and columns decreasing away from the diagonal. The corresponding circular order is defined as a perfect order. We have shown with a probabilistic model that perfect order is quite robust against lateral transfer and crossover (Thuillard, 2007). The search for the order minimizing a measure of the deviation from perfect order can be efficiently done with a multi-resolution algorithm (Thuillard, 2001, 2007). The method has been tested on SSU rRNA data for Archaea. The matrix with the best order corresponds quite well to a Robinson matrix. In this article, the minimum contradiction approach is further developed and applied to whole genome phylogenies.

With the availability of complete genomes, many methods have been proposed to determine the evolution of whole genomes (For reviews see Galperin et al. 2006; Delsuc et al. 2005; Henz et al. 2005). The construction of trees from whole genomes has proved over recent years to be a quite difficult task. This is mainly because of the very limited number of genes shared by Archaea, Eukaryota and Bacteria. Furthermore, gene evolution can sometimes be very different from species evolution. The main difficulty consists in finding a good operator to estimate the distance between genomes. Distances have been estimated with measures based on gene order or arrangement (Wolf et al. 2002; Wang et al. 2006), gene content (Fitz-Gibbon and House, 1999; Snel et al. 1999; Korbel et al. 2002), protein domain organization (Fukami-Kobayashi et al. 2007; Yang et al. 2005), folds (Lin and Gerstein, 2007), combining the information from many genes in a supertree or a superdistance (Dutihl et al. 2007 for a comparative study) or using a local alignment search tool such as Blast (Kunin et al. 2005b; Clarke et al. 2002). Among genome distances obtained with Blast, the genome conservation (Kunin et al. 2005b) has furnished some of the best trees up to date, if the quality of a whole genome phylogeny is measured by its concordance to broadly accepted classifications. The genome conservation estimates the distance between two taxa using the sum of BlastP reciprocal best hits between two genomes. The method is capable of quite correctly recovering all main phyla. At the phylum level, the evolution of the different genes is sufficiently similar to form a distinct cluster. The main uncertainties in whole genome phylogenies are on the relationships between phyla. Different evolution rates of the genes, gene losses or duplications, lateral gene transfer may result into large deviations of the distance matrix from a tree topology. In this context, minimum contradiction matrices can furnish information not contained in a single tree or a split network.

The paper is organized as follows. After introducing minimum contradiction matrices in section 2 and their connection to Robinson matrices and Kalmanson inequalities, section 3 explains why the identification of deviations from perfect order is a useful complement to phylogenetic studies. Section 4 presents an algorithm to search for the order minimizing a measure of the deviation from perfect order over all taxa. This order can be interpreted as an average best order over all reference taxa *Y_i_*_,_ *_j_^N^* (*N* = 1, *…*, *n*). The algorithm is applied in section 5 to distance matrices for whole genome phylogenies obtained with the genome conservation method.

## 2. Circular Order and the Minimum Contradiction Approach

### 2.1. Definitions

Let us start by recalling a number of definitions that are necessary to introduce the notion of circular order. A graph G is defined by a set of vertices *V*(*G*) and a set of edges *E*(*G*). Let us write *e*(*x*, *y*), the edge between the two vertices *x* and *y*. In a graph *G*, a path *P* between two vertices *x* and *y* is a sequence of non-repeating edges *e*(*x*_1_, *z*_1_), *e*(*z*_1_, *z*_2_), …, *e*(*z_i_*, *y*) connecting *x* to *y*. The degree of a vertex *x* is the number of edges *e* ∈ *E*(*G*) to which *x* belongs. A leaf *x* of a graph is a vertex of degree one. A vertex of degree larger than one is called an internal vertex.

A valued X-tree *T* is a graph with *X* as its set of leaves and a unique path between any two distinct vertices *x* and *y*, with internal vertices of at most degree 3. The distance *d* between leaves satisfies the classical triangular inequality

(1) d(x,z)≤d(x,y)+d(y,z) for all x,y,z∈X

with *d*(*x*, *y*) representing the sum of the weights on the edges of *T* in the path connecting *x* and *y*.

A central problem in phylogeny is to determine if there is an *X*-tree *T* and a real-valued weighting of the edges of *T* that fits a dissimilarity matrix *δ*. Typically, a dissimilarity matrix *δ* corresponds to an estimation of the pairwise distance *d*(*x_i_*, *x_j_*) between all elements in *X*. A necessary and satisfactory condition for the existence of a unique tree is that the dissimilarity matrix *δ* satisfies the so-called 4-point condition (Bunemann, 1971). For any four elements in *X*, the 4-point condition requires that

(2) $$\begin{array}{c} \delta (x_{i},x_{k})+\delta (x_{j},x_{n})\leq \text{max}(\delta (x_{i},x_{j})\\ +\delta (x_{k},x_{n}),\delta (x_{i},x_{n})+\delta (x_{j},x_{k})). \end{array}$$

### 2.2. Circular order and Kalmanson inequalities

Consider a planar representation of a tree *T* or a split network S. A circular order corresponds to an indexing of the n leaves according to a circular (clockwise or anti-clockwise) scanning of the leaves (Barthélemy and Guénoche, 1991; Makarenkov and Leclerc, 1997, 2000; Yushmanov, 1984).

**Figure 1:**
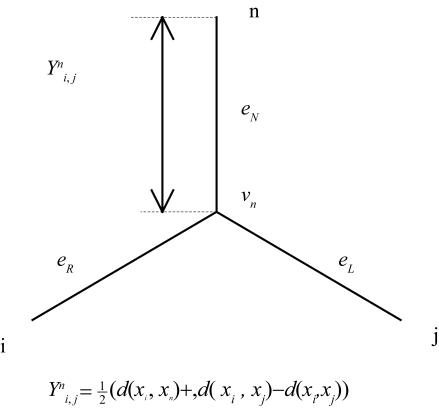
The distance matrix *Y_i, j_^n^* corresponds to the distance between the leaf *n* and the path *P*(*i*, *j* ).

In an X-tree, a circular order has the property that for any integer *k* (modulo *n*), all the branches on the path *P*(*x_k_*, *x_k_*_+1_) between *x_k_* and *x_k_*_+1_ correspond to the left branch (or right branch if anti-clockwise). A circular order can be obtained by considering the distance matrix *Y_i_*_,_ *_j_^n^*. As illustrated in Figure 1, the matrix element *Y_i_*_,_ *_j_^n^* = ½ (*d*(*x_i_*, *x_n_*) + *d*(*x_j_*, *x_n_*) − *d*(*x_i_*, *x_j_*)) corresponds to the distance between a reference leaf *n* and the path *P*(*x_i_*, *x_j_*). A circular order can be computed by ordering the distance matrix *Y_i_*_,_ *_j_^n^* so that it fulfils the inequalities defining a perfect order

(3a) $$Y_{i,j}^{n}\geq Y_{i,k}^{n},Y_{k,j}^{n}\geq Y_{k,i}^{n}\;(i\leq j\leq k<n).$$

The above inequalities characterize also a Robinson matrix (Christopher et al. 1996; Thuillard, 2007). Using the definition of *Y_i_*_,_ *_j_^n^* the inequalities become

$$d(x_{j},x_{n})+d(x_{l},x_{k})\geq d(x_{k},x_{n})+d(x_{i},x_{j})$$

and

(3b) $$\begin{array}{l} d(x_{j},x_{n})+d(x_{k},x_{i})\geq d(x_{i},x_{n})\\ +d(x_{k},x_{j})\;(i\leq j\leq k<n). \end{array}$$

These inequalities have a similar form to the 4-point condition (2) and are known as the Kalmanson inequalities.

### 2.3. Minimum contradiction matrix

In real applications, the distance matrix *Y_i_*_,_ *_j_^n^* does often only partially fulfill the inequalities corresponding to a perfect order. The contradiction on the order of the taxa can be defined as

(4) $$\begin{array}{l} C=\sum_{\begin{array}{c} k>j\geq i\\ i,j,k\neq n \end{array}}{\left(\text{max}\left(\left(Y_{i,k}^{n}-Y_{i,j}^{n}\right),0\right)\right)}^{\beta }\\ +\sum_{\begin{array}{c} k\geq j>i\\ i,j,k\neq n \end{array}}{\left(\text{max}\left(\left(Y_{i,k}^{n}-Y_{j,k}^{n}\right),0\right)\right)}^{\beta }. \end{array}$$

The best order of a distance matrix is, per definition, the order minimizing the contradiction. The ordered matrix *Y_i_*_,_ *_j_^n^* corresponding to the best order is defined as the minimum contradiction matrix for the reference taxon *n*.

For a perfectly ordered X-tree, the contradiction *C* is zero. A tree with a low contradiction value *C* is a tree that can be trusted, while a high contradiction value *C* is the indication of a distance matrix deviating significantly from an X-tree.

## 3. Why Perfect Order is an Important Property?

Kalmanson inequalities are at the center of a number of important results relating convexity (Kalmanson, 1975), the Traveling Salesman Problem (TSP) (Deineko et al. 1995; Korostensky and Gonnet, 2000), phylogenetic trees and networks (Christopher et al.1996; Dress and Huson, 2004). Let us explain why perfect order is an important property.

– If the error on the distance in an X-tree is not greater than *x*_min_/2 with *x*_min_ the shortest edge on the tree, then the Neighbor-Joining algorithm will recover the correct tree topology and Kalmanson inequalities hold (Atteson, 1999; Korostensky and Gonnet, 2000).
– If a distance matrix d fulfills Kalmanson inequalities, then the distance matrix can be exactly represented by a split network (Bandelt and Dress, 1992).
– If Kalmanson inequalities are fulfilled, then the tour (1, 2, *…, n*) corresponds to a solution of the Traveling Salesman Problem (Christopher et al. 1996).

The last result can be demonstrated starting from the sum $\sum_{i=1,\ldots ,n-2}Y_{i,i+1}^{n}$. When Kalmanson inequalities are fulfilled, the sum $\sum_{i=1,\ldots ,n-2}Y_{i,i+1}^{n}$ is maximized. As *Y_i_^n^*_,_ *_i_*_+1_ ≥ *Y_i_^n^*_,_ *_i_*_+_*_m_* (*i* + *m* ≤ *n*, *m* > 1). Developing $\sum_{i=1,\ldots ,n-2}Y_{i,i+1}^{n}$, one gets $\sum_{i=1,\ldots ,n-1}Y_{i,i+1}^{n}=\sum_{i=1,\ldots ,n}d_{i,n}-1/2\cdot (d_{1,n}+\sum_{i=1,\ldots ,n-1}d_{i,i+1})$. The first sum $\sum_{i=1,\ldots ,n}d_{i,n}$ is independent of the order and one concludes that a perfect order minimizes $d_{1,n}+\sum_{i=1,\ldots ,n-1}d_{i,i+1}$. The tour (1, 2, *…*, *n*) is therefore a solution of the TSP.

The solution to the TSP has the Master Tour property (Deineko et al. 1995). A Master Tour is a solution of the TSP with the property that the optimal tour restricted to a subset of points is also a solution of the reduced TSP. This result follows directly from the inequalities for perfect order *Y_i_*_,_ *_j_^n^* ≥ *Y_i_*_,_ *_k_^n^*, *Y_k_*_,_ *_j_^n^* ≥ *Y_k_*_,_ *_i_^n^* (*i* ≤ *j* ≤ *k* < *n*). Any restriction of a perfectly ordered distance matrix *Y_i_*_,_ *_j_^n^* to a subset of taxa is perfectly ordered and consequently is a solution to the reduced TSP. In contrast to this result, one finds with numerical experiments that, if the minimum contradiction matrix does not fulfill the inequalities for perfect order, the best order is not always preserved when a number of taxa are removed. The order minimizing the contradiction over n taxa does not always minimize the contradiction when restricted to a subset of taxa. It follows that one cannot exclude that the topology of a tree or a split network may change when taxa contradicting perfect order are removed. Deviations from perfect order correspond to problematic regions that have to be interpreted very carefully. For that reason we suggest that minimum contradiction matrices are a useful complement to any distance-based phylogeny.

## 4. Searching for the Best Order in Whole Genome Phylogenies

### 4.1. Fast algorithm to search for the best order

The choice of the reference taxon *n* in *Y_i_*_,_ *_j_^n^* can significantly influence the best order, when the distance matrix cannot be perfectly ordered. For that reason, an average best order is determined by minimizing the contradiction over all reference taxa.

The contradiction over all n reference taxa is given by

(5) $$\begin{array}{l} C=\sum_{m=1,\ldots ,n}(\sum_{\begin{array}{c} k>j\geq i\\ i,j,k\neq n \end{array}}{\left(\text{max}\left(\left(Y_{i(m),k(m)}^{n(m)}-Y_{i(m),j(m)}^{n(m)}\right),0\right)\right)}^{\beta }\\ +\sum_{\begin{array}{c} k\geq j>i\\ i,j,k\neq n \end{array}}{\left(\text{max}\left(\left(Y_{i(m),k(m)}^{n(m)}-Y_{j(m),k(m)}^{n(m)}\right),0\right)\right)}^{\beta }) \end{array}$$

with *i*(*m*) = mod(*m* + *i*_0_ − 2, *n*) + 1; *j*(*m*) = mod(*m* + *j*_0_ − 2, *n*) + 1, *n*(*m*) = *n*_0_ −*m* + 1 and *β* = 2.

The best order is the order (1, …, *i*_0_, …, *j*_0_, …, *n*_0_) minimizing the contradiction. The computation of the contradiction requires O(*n*^4^) operations. For a large ensemble of taxa, the computational cost may become quite high. We will therefore introduce below an algorithm requiring only O(*n*^3^) operations to compute a (slightly different) measure of the contradiction.

**Figure 2:**
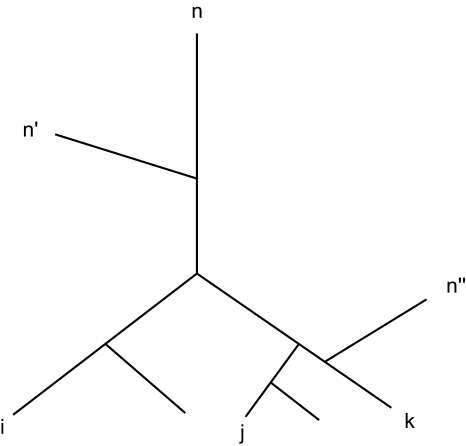
The inequalities *Y_i_*_,_ *_j_^n^* ≥ Y*_i, k_^n^* are fulfilled for any reference vertex *n* with *n* ≥ *k* or *n* ≤ *i*.

Let us start by considering an X-tree and the 3 vertices *i*, *j*, *k* as in Figure 2. The distance matrix fulfills the inequalities for perfect order. The order between the vertices *i*, *j*, *k* is preserved for any reference vertex not in the interval (*i*, *k*) and the inequalities *Y_i_*_,_ *_j_^n^* ≥ *Y_i_*_,_ *_k_^n^* and *Y_k_*_,_ *_j_^n^* ≥ *Y_k_*_,_ *_i_^n^* *n* = 1, …, *i*, *k*, …, *N* hold. The inequalities can be summed up over all *n* and one obtains two new inequalities:

(6a) Sa(i,j)≥Sa(i,k)         (i≤j≤k)

(6b) Sb(i,j)≥Sb(i,k)         (i≤j≤k)

With

(6c) $$Sa(i,j)=\sum_{n=1,\ldots ,i}Y_{i,j}^{n};\;\;\;Sb(i,j)=\sum_{n=k,\ldots ,N}Y_{i,j}^{n}$$

If the contradiction *c_i_*_,_ *_j_* between the vertices *i*, *j* is defined as the sum of two terms

(7a) $$c_{i,j}=ca_{i,j}+cb_{i,j}\;\text{with}$$

(7b) $$ca_{i,j}=\sum_{k\geq j\geq i}\text{max}(0,{\left(Sa(i,k)-Sa(i,j)\right)}^{2})$$

(7c) $$cb_{i,j}=\sum_{k\geq j\geq i}\text{max}(0,{\left(Sb(i,k)-Sb(i,j)\right)}^{2})$$

then the best order is the order minimizing $c=\sum_{\begin{array}{l} i=1,\ldots ,N\\ j\geq i \end{array}}ca_{i,j}+cb_{i,j}$. Computing the contradiction requires O(*n*^3^) operations (As the computation of the contradiction is the most computer-intensive, the algorithm requires approximately n times less computing time than the O(*n*^4^) algorithm).

The quantities *Sa* and *Sb* in Eq. (6) can be related to the NJ algorithm. For 3 consecutive vertices (*i*, *j* = *i* + 1, *k* = *i* + 2), Eq. (6a) can be written, assuming perfect order, as

(8) $$\sum_{n\neq i,i+1,i+2}Y_{i,i+1}^{n}\geq \sum_{n\neq i,i+1,i+2}Y_{i,i+2}^{n}$$

Writing $r_{i}=\sum_{n=1,\ldots ,N}d(x_{i},x_{n})$ and *S_i, j_* = *r_i_* + *r_j_* − (*N* – 2). *d*(*x_i_*, *x_j_*) one obtains

(9) $$S_{i,j+1}-S_{i,i+2}\geq 0.$$

The value *S_i_*_,_ *_j_* is central to the NJ algorithm (Saitou and Nei, 1987; Gascuel and Steel, 2006 ). Two vertices *i*, *j* are joined by the NJ algorithm, if they maximize *S* (i.e. max(*S*) = *S_i_*_,_ *_j_*). From the above discussion, it seems natural to initialize the search for the best order on the NJ tree. The search for the best order of *Y_i_*_,_ *_j_^n^* is initialized with the NJ algorithm and a small supplementary procedure that we describe below. Given two vertices a and b that are joined by the NJ algorithm and the leaves *a*_1_, *a*_2_, …, *a_i_* (*resp. b*_1_, *b*_2_, …, *b_j_*) that have the vertex *a* (resp. *b*) as first ancestor. The best order of the leaves is chosen so as to minimize the contradiction among 4 possibilities: (*ab*, *āb*, *ab̄*, *āb* with *ab* the order *a*_1_, *a*_2_, …, *a_i_*, *b*_1_, *b*_2_, …, *b_j_* and *ā* the inversed order *a_i_*, *a_i_*_–1_, …, *a*_1_. Once the order is optimized over the NJ tree, the best order is refined with a multiresolution search algorithm (Thuillard, 2001, 2007).

### 4.2. Similarity matrix for whole genomes phylogenies

For whole genome phylogenies, the search for appropriate measures to estimate the evolutionary distance between taxa is still the subject of significant research efforts (Korbel et al. 2002; Kunin et al. 2005b; Yang et al. 2005; Fukami-Kobayashi, 2007). Distance matrices obtained from BlastP scores have been quite successful to generate good trees. The similarity score obtained with BlastP programs can be given a probabilistic interpretation. The statistics of high scoring segments in the absence of gaps tends to an extreme value distribution (Karlin and Altschul, 1990). The probability *P* of finding at least a high scoring segment is well approximated, for small values of *P*, by the formula *P* = *m*_1_·*m*_2_·2^−^*^Score^* with *m*_1_, *m*_2_ the length of the 2 sequences. It follows that *Score* = −log_2_ *P* + log_2_(*m*_1_·*m*_2_). Defining the distance *d* between two sequences as *d* = −*Score* and assuming equal lengths one has *d* = log_2_(*P*/*m*^2^). Using that definition, the distance matrix *Y_i_*_,_ *_j_^n^* becomes for 3 sequences

(10) $$Y_{i,j}^{n}=1/2\cdot \left({\text{log}}_{2}\left(\frac{P(i|n)\cdot P(j|n)}{P(i|j)\cdot m^{2}}\right)\right).$$

The log term has the form of a mutual information and furnishes a measure of the similarity of the genomes i and j in reference to the genome *n*.

Different approaches have been proposed to normalize the distance matrix using the marginal entropy (Kraskov et al. 2005), the self-score (Kunin et al. 2005b), Korbel normalization (Korbel et al. 2002) or the average score. The normalization by the self-score in the genome conservation gives some of the best results. It is based on a nonlinear weighted sum of the BlastP scores. The gene conservation method computes the distance between two taxa by normalizing the sum of reciprocal best hits between genome *i* and *j* by the self-score. The effect of duplication is limited by using only reciprocal best hits. The normalization by the self-score is important to correct, at least partially, the effect of different genome sizes. The genome conservation similarity matrix is given by

(11) $$S_{i,j}=\text{min}\left(\sum \left(i,j\right),\sum \left(j,i\right)\right)/\text{min}\left(\sum \left(i,i\right),\sum \left(j,j\right)\right)$$

with ∑ (*i*,*j*) the sum of reciprocal best hits between the genomes of the two taxa.

## 5. Whole Genome Phylogenies

### 5.1. Search for the best average order

**Figure 3:**
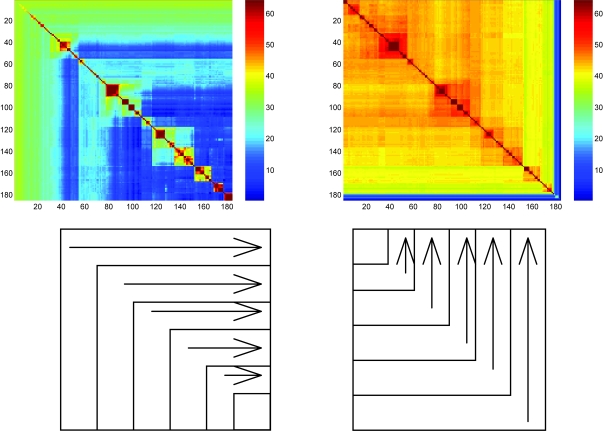
Minimum contradiction matrices corresponding to the best order found after optimization with Eq. 6,7. The contradiction is minimized over the lines of the matrix $Sa(i,j)=\sum_{n=1,\ldots ,i}Y_{i,j}^{n}$ (left) and the columns $Sb(i,j)=\sum_{n=k,\ldots ,N}Y_{i,j}^{n}$ (right).

The algorithms described in section 4 have been used to search for the best order. The distance matrix was computed using the data furnished by the genome phylogeny server (Kunin et al. 2005b) obtained with an e-value cut-off set to 10^−10^. The contradiction is significantly lower with the score (1 – *S_i_*_,_*_j_*) than with the logarithm of the score. Figure 3 shows the best order after optimization with the algorithms described in section 4 followed by 5000 steps of the multiresolution search algorithm using Eq. (7) to compute the contradiction.

Table 1 gives the order of the different taxa corresponding to the best order. Archaea and Eukaryota are grouped into two adjacent clusters of taxa. One observes, for Bacteria, that all the members of a class or a phylum are neighbors. All proteobacteria (together with Aquifex?) are grouped together. The best order obtained with the minimum contraction approach differs from the NJ tree on the following aspect: all spirochetes and δ-proteobacteria form a cluster. This is not the case of the NJ tree.

### 5.2. Interpreting minimum contradiction matrices

**Figure 4:**
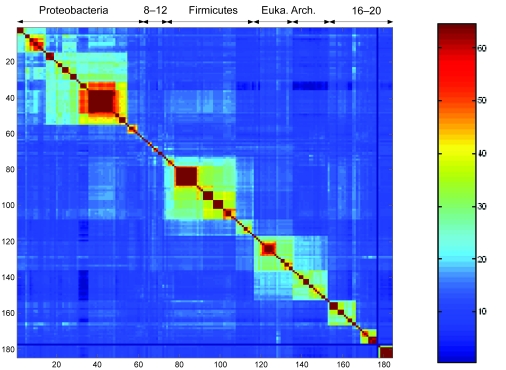
Distance matrix *Yi, j^n^* using the best order in Figure 3 and *Pirellula* (taxon 177) as reference taxon.

This article focus on the mathematical aspects of Minimum Contradiction Matrices. We will limit the discussion to 3 examples showing how to interpret Minimum Contradiction Matrices. The matrix *Y_i_*_,_ *_j_^n^* can be imaged for different reference taxa using the best order of Figure 3 given in the annex. Figure 4 shows the matrix *Y_i_*_,_ *_j_^n^* using *Pirellula* (taxa 177) as reference taxa. The scale on the right of the figure gives the color code used to represent *Y_i_*_,_ *_j_^n^* after rescaling. The minimum value of *Y_i_*_,_ *_j_^n^* corresponds to dark blue, while the largest values are coded red. Low values of *Y_i_*_,_ *_j_^n^* are associated to two vertices (*i*, *j*) having a first common ancestor vertex close to the reference taxa. A cluster of adjacent taxa with large values (red cluster) can be interpreted as a group of close taxa. One observes that Archaea and Eukaryota are not only adjacent but form also a cluster.

The best order in Figure 3 is obtained by minimizing the contradiction using all taxa as reference vertex at least once. The best order is therefore a kind of “average” best order. The matrix *Y_i, j_^n^* (resp. $\sum_{n=n_{1},\ldots ,n_{k}}Y_{i,j}^{n}$) with n corresponding to a unique taxon (resp. a group of taxa belonging to some phylum) allows the identification of large contradictions from the best order. These contradictions can often be specifically related to the reference taxon. A loss of a gene, a lateral gene transfer or a crossover in the reference taxon modifies all elements of the distance matrix *Y_i, j_^n^*. A similar perturbation on a taxon that is not a reference taxon affects at most the row and the column corresponding to that taxon.

**Figure 5:**
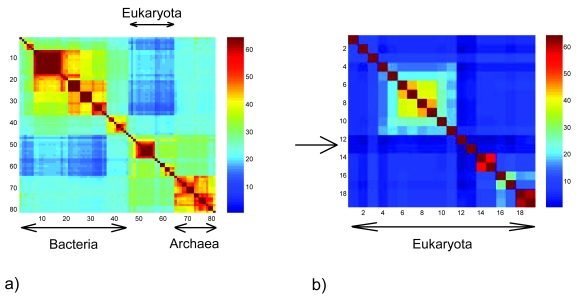
Distance matrix *Yi, j^n^* for a) Rickettsiales (Taxa 1–4) as reference taxa and taxa 72–152 in Figure 3. b) Eukaryota using Cyanobacteria as reference taxa. The arrow points to *Arabidopsis* and *Cyanidioschyzon*.

Many contradictions in Figure 5 can be associated to well accepted endosymbiotic events (Chloroplasts in plants or mitochondria in Eukaryota). Figure 5a shows *Y_i, j_^n^* for Archaea, Eukaryota and some Bacteria (Taxa 72–116) using Rickettsiales (Taxa 1–4 in annex) as reference taxa. The average best order is used to order the taxa. Contradictions on the order of the taxa are identified by looking for regions with *Y_i, j_^n^* increasing away from the diagonal (i.e. *Y_i, j_^n^* < *Y_i, j_^n^*, *i* < *j* < *k* < *n*). Contradictions are observed for *i* = Bacteria (without Mycoploasma) *j* = Eukaryota. One observes that *Y_i, j_^n^* decreases away from the diagonal except between Eukaryota and Archaea (dark blue compared to light blue for Archaea). This result is, at first glance, somewhat surprising. Similar values of *Y_i, j_^n^* for Archaea and Eukaryota are expected when *i*, *n* correspond to Bacteria. The low values for Eukaryota can be explained by a lateral transfer between the Rickettsiales and Eukaryota. We have shown with a probabilistic model (Thuillard, 2007) that a lateral transfer between the reference taxa and some taxa reduces the expected values of *Y_i, j_^n^* for those taxa. In this model, the expected value *Ŷ_i, j_^n^* after an α-lateral transfer is given by *Ŷ*_*E*_1_*,E*_2__*^R^* = (1 − α) · *Y*_*E_1_,E_2_*_*^R^* + α · *Y_R_1_,R_2__^R^* ≤ *Y_E_1_,E_2__^R^* with α the proportion of the genome laterally transferred (α ≤ 1) from the reference taxa *R*, and *R_1_*, *R_2_* the laterally transferred sequence after further evolution into the Eukaryota genomes *E*_1_, *E*_2_. The observed contradiction and the small values of *Y_i, j_^n^* for Eukaryota are consistent with a lateral transfer between the reference taxa (Rickettsiales) and Eukaryota. Let us recall here that mitochondria are believed to be the result of an endosymbiotic event involving Rickettsia (Timmis et al. 2004), an event that resulted also into the transfer of some Rickettsia genes into the nucleus of the host.

Figure 5b shows the distance matrix using all Cyanobacteria as reference taxa. The elements associated to *Arabidopsis* and *Cyanidioschyzon* have lower values than both adjacent lines (resp. columns). The observed contradictions for *Arabidopsis* and *Cyanidioschyzon merolae* (a plant and a red alga) may be explained by the many genes that are found in both Cyanobacteria and plants/red alga but absent in other Eukaryota, a hypothesis that is supported by the small value of the distance between Cyanobacteria and (*Arabidopsis*, *Cyanidioschyzon*). Chloroplasts in plants and red alga are generally considered to have originated as endosymbiotic Cyanobacteria. The low values of *Y_i, j_^n^* for *i* = *Arabidopsis*, *Cyanidioschyzon* are compatible with the hypothesis that some Cyanobacteria genes have been transferred into the host.

## Conclusions

For an X-tree or a split network the minimum contradiction matrix $Y_{i,j}^{n}=\frac{1}{2}(d(x_{i},x_{n})+d(x_{j},x_{n})-d(x_{i},x_{j}))$ fulfills all the inequalities defining perfect order (i.e. *Y_i, j_^n^* ≥ *Y_i, k_^n^*, *Y_k, j_^n^* , ≥ *Y_k_^n^*, *i* ≤ *j* ≤ *k* ≤*n*). In real applications a number of taxa may typically be in contradiction to the inequalities for perfect order. In that case, the Master Tour property does not hold. It follows that the removal or the addition of taxa in contradiction to the inequalities may change the topology of the associated NJ tree or split network.

An average best order can be obtained by searching for the best circular order over *Y_i, j_^n^* (*N* 1, *…*, *n*). The matrix *Y_i, j_^n^* can be used to localize a problematic taxon, as large deviations from the average best order are often related to the reference taxon *n*. This approach was applied to whole genome phylogenies using distances computed with the genome conservation method. Several large deviations from the average best order were found to correspond to well-documented evolutionary events.

## Figures and Tables

**Table 1:** Best Order (Fig. 3, 4).

1. α-Proteobacteria	1–14
2. γ-Proteobacteria	15–18
3. β-Proteobacteria	19–29
4. γ-Proteobacteria	30–54
5. ɛ-Proteobacteria	55–59
6. Aquificae	60
7. δ-Proteobacteria	61–63
8. Chlorobi	64
9. Bacteroidetes	65–66
10. Spirochetes	67–71
11. Thermotogae	72
12. Fusobacteria	73
13. Firmicutes	74–116
14. Eukaryota	117–135
15. Archaea	136–152
16. Actinobacteria	153–166
17. Deinococcus-Thermus	167–168
18. Cyanobacteria	169–176
19. Planctomycetes	177
20. Chlamydiae	178–184

(see annex for detailed list of taxa).

## Annex: List of Taxa Corresponding to the Best Order of Figure 3

1. RTYP-144-01-Rickettsia typhi ATCC VR-144
2. RPRO-MAD-01-Rickettsia prowazekii Madrid E
3. RCON-MAL-01-Rickettsia conorii str. Malish 7
4. WPIP-WME-01-Wolbachia pipientis wMeI
5. BJAP-USD-01-Bradyrhizobium japonicum USDA110
6. RPAL-009-01-Rhodopseudomonas palustris CGA009
7. BQUI-TOU-01-Bartonella quintana Toulouse
8. BHEN-HOU-01-Bartonella henselae Houston-1
9. BMEL-M16-01-Brucella melitensis M16
10. BSUI-133-01-Brucella suis str. 1330
11. ATUM-C58-01-Agrobacterium tumefaciens C58
12. SMEL-102-01-Sinorhizobium meliloti strain 1021
13. MLOT-MAF-01-Mesorhizobium loti MAFF303099
14. CCRE-XXX-01-Caulobacter crescentus CB15
15. XAXO-306-02-Xanthomonas axonopodis pv. citri str. 306
16. XCAM-AT3-01-Xanthomonas campestris pv. campestris ATCC33913
17. XFAS-XPD-01-Xylella fastidiosa PD
18. XFAS-9A5-01-Xylella fastidiosa 9a5c
19. NEUR-718-01-Nitrosomonas europaea ATCC19718
20. CVIO-472-01-Chromobacterium violaceum ATCC 12472
21. NMEN-Z24-01-Neisseria meningitidis Z2491
22. NMEN-MC5-01-Neisseria meningitidis MC58
23. BPER-251-01-Bordetella pertussis NCTC-13251
24. BBRO-252-01-Bordetella bronchiseptica NCTC- 13252
25. BPAR-253-01-Bordetella parapertussis NCTC-13253
26. RSOL-XXX-01-Ralstonia solanacearum
27. PSYR-DC3-01-Pseudomonas syringae pv. tomato DC3000
28. PPUT-KT2-01-Pseudomonas putida KT2440
29. PAER-PAO-01-Pseudomonas aeruginosa PAO1
30. CBUR-RSA-01-Coxiella burnetii RSA 493
31. WGLO-BRE-01-Wigglesworthia glossinidia brevipalpis
32. BAPH-XSG-01-Buchnera aphidicola SG
33. BUCH-APS-01-Buchnera sp. APS
34. BAPH-XBP-01-Buchnera aphidicola Bp
35. BFLO-XXX-01-Blochmannia floridanus
36. ECAR-043-01-Erwinia carotovora subsp. atroseptica SCRI1043
37. YPES-CO9-01-Yersinia pestis CO92
38. YPES-KIM-01-Yersinia pestis KIM
39. SFLE-457-01-Shigella flexneri 2457T
40. SFLE-301-01-Shigella flexneri str. 301
41. ECOL-RIM-01-Escherichiacoli 0157H7 RIMD0509952
42. ECOL-EDL-01-Escherichia coli O157H7 EDL933
43. ECOL-MG1-01-Escherichia coli MG1655
44. ECOL-CFT-01-Escherichia coli CFT073
45. SENT-TY2-01-Salmonella enterica Ty2
46. SENT-CT1-02-Salmonella enterica serovar Typhi CT18
47. SENT-LT2-01-Salmonella enterica serovar Typhimurium LT2
48. PLUM-TO1-01-Photorhabdus luminescens TTO1
49. HINF-KW2-01-Haemophilus influenzae KW20
50. PMUL-PM7-01-Pasteurella multocida Pm70
51. VCHO-N16-01-Vibrio cholerae El Tor N16961
52. VVUL-YJ0-01-Vibrio vulnificus YJ016
53. VPAR-RIM-01-Vibrio parahaemolyticus RIMD2210633
54. SONE-MR1-01-Shewanella oneidensis MR1
55. CJEJ-NCT-01-Campylobacter jejuni NCTC 11168
56. HPYL-266-01-Helicobacter pylori 26695
57. HPYL-J99-01-Helicobacter pylori J99
58. HHEP-449-01-Helicobacter hepaticus ATCC51449
59. WSUC-740-01-Wolinella succinogenes strain DSM 1740
60. AAEO-VF5-01-Aquifex aeolicus VF5
61. GSUL-PCA-01-Geobacter sulfurreducens PCA
62. DVUL-HIL-01-Desulfovibrio vulgaris str. Hildenborough
63. BBAC-100-01-Bdellovibrio bacteriovorus HD100
64. CTEP-TLS-01-Chlorobium tepidum TLS
65. BTHE-VPI-01-Bacteroides thetaiotaomicron VPI-5482
66. PGIN-W83-01-Porphyromonas gingivalis W83
67. BBUR-B31-01-Borrelia burgdorferi B31
68. TDEN-405-01-Treponema denticola ATCC 35405
69. TPAL-NIC-01-Treponema pallidum Nichols
70. LINT-566-01-Leptospira interrogans str. 56601
71. LINT-130-01-Leptospira interrogans L1-130
72. TMAR-MSB-01-Thermotoga maritima MSB8
73. FNUC-ATC-01-Fusobacterium nucleatum ATCC 25586
74. TTEN-MB4-01-Thermoanaerobacter tengcongensis MB4
75. CTET-E88-01-Clostridium tetani E88
76. CPER-X13-01-Clostridium perfringens str. 13
77. CACE-ATC-01-Clostridium acetobutylicum ATCC 824
78. LLAC-IL1-01-Lactococcus lactis IL1403
79. SMUT-UA1-01-Streptococcus mutans UA159
80. SAGA-260-01-Streptococcus agalactiae 2603 V/R
81. SAGA-NEM-01-Streptococcus agalactiae NEM316
82. SPYO-SF3-01-Streptococcus pyogenes M1 SF370
83. SPYO-MGA-01-Streptococcus pyogenes M18 MGAS8232
84. SPYO-XM3-01-Streptococcus pyogenes M3 MGAS315
85. SPYO-SSI-01-Streptococcus pyogenes M3 SSI-1
86. SPYO-394-01-Streptococcus pyogenes MGAS10394
87. SPNE-TIG-01-Streptococcus pneumoniae TIGR4
88. SPNE-XR6-01-Streptococcus pneumoniae R6
89. EFAE-V58-01-Enterococcus faecalis V583
90. LPLA-WCF-01-Lactobacillus plantarum WCFS1
91. LJOH-533-01-Lactobacillus johnsonii NCC 533
92. LINN-CLI-01-Listeria innocua CLIP 11262
93. LMON-365-01-Listeria monocytogenes F2365
94. LMON-858-01-Listeria monocytogenes H7858
95. LMON-854-01-Listeria monocytogenes F6854
96. LMON-EGD-01-Listeria monocytogenes EGD-e
97. SAUR-476-01-Staphylococcus aureus MSSA476
98. SAUR-MW2-01-Staphylococcus aureus MRSA MW2
99. SAUR-N13-01-Staphylococcus aureus MRSA N315
100. SAUR-MU5-01-Staphylococcus aureus VRSA Mu50
101. SAUR-252-01-Staphylococcus aureus MRSA252
102. BSUB-168-01-Bacillus subtilis 168
103. BANT-AME-01-Bacillus anthracis Ames
104. BCER-987-01-Bacillus cereus ATCC 10987
105. BCER-579-01-Bacillus cereus ATCC 14579
106. OIHE-HET-01-Oceanobacillus iheyensis HET831
107. BHAL-C12-01-Bacillus halodurans C-125
108. MMYC-G1T-01-Mycoplasma mycoides subsp. mycoi- des SC strain PG1
109. MMOB-63K-01-Mycoplasma mobile 163K
110. MPUL-UAB-01-Mycoplasma pulmonis UAB CTIP
111. UURE-SV3-01-Ureaplasma urealyticum serovar 3
112. MGEN-G37-01-Mycoplasma genitalium G-37
113. MPNE-M12-01-Mycoplasma pneumoniae M129
114. MGAL-RLO-01-Mycoplasma gallisepticum Rlow
115. MPEN-HF2-01-Mycoplasma penetrans HF2
116. PAST-XOY-01-Phytoplasma asteris OY
117. CPAR-TII-01-Cryptosporidium parvum typeII
118. PFAL-3D7-01-Plasmodium falciparum 3D7
119. ECUN-XXX-01-Encephalitozoon cuniculi
120. NCRA-XX3-01-Neurospora crassa
121. YLIP-B99-01-Yarrowia lipolytica CLIB99
122. AGOS-XXX-01-Ashbya gossypii
123. KLAC-210-01-Kluyveromyces lactis CLIB210
124. SCER-S28-01-Saccharomyces cerevisiae S288C
125. CGLA-138-01-Candida glabrata CBS138
126. DHAN-767-01-Debaryomyces hansenii CBS767
127. SPOM-XXX-01-Schizosaccharomyces pombe
128. ATHA-XXX-01-Arabidopsis thaliana
129. CMER-10D-01-Cyanidioschyzon merolae 10D
130. CBRI-XXX-01-Caenorhabditis briggsae
131. CELE-XXX-01-Caenorhabditis elegans
132. DMEL-XXX-02-Drosophila melanogaster
133. AGAM-PES-01-Anopheles gambiae PEST
134. HSAP-XXX-03-Homo sapiens v15.33.1
135. MMUS-XXX-02-Mus musculus
136. NEQU-N4M-01-Nanoarchaeum equitans Kin4-M
137. APER-XK1-01-Aeropyrum pernix K1
138. PAER-IM2-01-Pyrobaculum aerophilum IM2
139. STOK-XX7-01-Sulfolobus tokodaii str. 7
140. SSOL-XP2-01-Sulfolobus solfataricus P2
141. TACI-DSM-01-Thermoplasma acidophilum DSM1728
142. TVOL-GSS-01-Thermoplasma volcanium GSS1
143. PTOR-790-01-Picrophilus torridus DSM9790
144. PABY-GE5-01-Pyrococcus abyssi GE5
145. PHOR-OT3-01-Pyrococcus horikoshii OT3
146. MTHE-DEL-01-Methanobacterium thermoautotrophicum deltaH
147. MJAN-DSM-01-Methanococcus jannaschii DSM 2661
148. MKAN-AV1-01-Methanopyrus kandleri AV19
149. AFUL-DSM-01-Archaeoglobus fulgidus DSM4304
150. MMAZ-GO1-01-Methanosarcina mazei Go1
151. MACE-C2A-01-Methanosarcina acetivorans C2A
152. HALO-NRC-01-Halobacterium sp. NRC-1
153. BLON-NCC-01-Bifidobacterium longum NCC2705
154. MTUB-CDC-01-Mycobacterium tuberculosis CDC1551
155. MTUB-H37-01-Mycobacterium tuberculosis H37Rv
156. MBOV-AF2-01-Mycobacterium bovis AF2122/97
157. MLEP-XTN-01-Mycobacterium leprae TN
158. CEFF-YS3-01-Corynebacterium efficiens YS314T
159. CGLU-XXX-01-Corynebacterium glutamicum
160. CDIP-129-01-Corynebacterium diphtheriae NCTC13129
161. PACN-202-01-Propionibacterium acnes KPA171202
162. LXYL-B07-01-Leifsonia xyli subsp. xyli CTCB07
163. TWHI-TWI-01-Tropheryma whipplei Twist
164. TWHI-TW0-01-Tropheryma whipplei TW08/27
165. SCOE-A32-01-Streptomyces coelicolor A3
166. SAVE-XXX-01-Streptomyces avermitilis
167. TTHE-B27-01-Thermus thermophilus HB27
168. DRAD-XR1-01-Deinococcus radiodurans R1
169. GVIO-421-01-Gloeobacter violaceus PCC 7421
170. SYNE-PCC-01-Synechocystis sp. PCC6803
171. TELO-BP1-01-Thermosynechococcus elongatus BP-1
172. NOST-PCC-01-Anabaena sp. strain PCC 7120
173. PMAR-SS1-01-Prochlorococcus marinus SS120
174. PMAR-MED-01-Prochlorococcus marinus MED4
175. SYCC-WH8-01-Synechococcus sp. WH8102
176. PMAR-MIT-01-Prochlorococcus marinus MIT9313
177. PIRE-ST1-01-Pirellula sp. strain 1
178. PCHL-E25-01-Parachlamydia sp. UWE25
179. CCAV-GPI-01-Chlamydophila caviae GPIC
180. CPNE-J13-01-Chlamydia pneumoniae J138
181. CPNE-CWL-01-Chlamydia pneumoniae CWL029
182. CPNE-AR3-01-Chlamydia pneumoniae AR39
183. CTRA-SVD-01-Chlamydia trachomatis serovar D
184. CTRA-MOP-01-Chlamydia trachomatis MoPn

## References

[b1-ebo-4-237] Atteson K (1999). The Performance of Neighbor-Joining Methods of Phylogenetic Reconstruction. Algorithmica.

[b2-ebo-4-237] Barthélemy JP, Guénoche A (1991). Trees and proximity representations.

[b3-ebo-4-237] Bandelt HJ, Dress A (1992). Split decomposition: a new and useful approach to phylogenetic analysis of distance data. Molecular Phylogenetic Evolution.

[b4-ebo-4-237] Bertrand P, Diday E (1985). A visual representation of the compatibility between an order and a dissimilarity index: the pyramids. Computational Statistics Quarterly.

[b5-ebo-4-237] Bryant D, Moulton V (2004). Neighbor-Net: an agglomerative method for the construction of phylogenetic networks. Molecular Biology and Evolution.

[b6-ebo-4-237] Buneman P, Hodson FR, Kendall DG, Tautu P (1971). The recovery of trees from measures of dissimilarity. Mathematics in the Archaeological and Historical Sciences.

[b7-ebo-4-237] Christopher GE, Farach M, Trick MA (1996). The structure of circular decomposable metrics. In European Symposium on Algorithms (ESA)’96, Lectures Notes in Computer Science.

[b8-ebo-4-237] Clarke GDP, Beiko R, Ragan MA, Charlebois RL (2002). Inferring genome trees by using a filter to eliminate phylogenetically discordant sequeneces and a distance matrix based on mean normalized BlastP scores. Journal of Bacteriology.

[b9-ebo-4-237] Delsuc F, Brinkmann H, Philippe H (2005). Phylogenomics and the reconstruction of the tree of life. Nature Reviews Genetics.

[b10-ebo-4-237] Deineko V, Rudolf R, Woeginger G (1995). Sometimes traveling is easy: the master tour problem, Institute of Mathematics, SIAM. Journal on Discrete Mathematics.

[b11-ebo-4-237] Dress A, Huson D (2004). Constructing split graphs. IEEE Transactions on Computational Biology and Bioinformatics.

[b12-ebo-4-237] Dutilh BE, Noort V, Heijden RTJM, Boekhout T, Snel B, Huynen MA (2007). Assessment of phylogenomic and orthology approaches for phylogenetic inference. Bioinformatics.

[b13-ebo-4-237] Fitz-Gibbon ST, House CH (1999). Whole genome-based phylogenetic analysis of free-living microorganisms. Nucleic Acids Research.

[b14-ebo-4-237] Fukami-Kobayashi K, Minezaki Y, Tateno Y, Nishikawa K (2007). A tree of life based on protein domain organizations. Molecular Biology and Evolution.

[b15-ebo-4-237] Galperin MY, Kolker E (2006). New metrics for comparative genomics. Current Opinion in Biotechnology.

[b16-ebo-4-237] Gascuel O, Steel M (2006). Neighbor-joining revealed. Molecular Biology and Evolution.

[b17-ebo-4-237] Grünewald S, Forslund K, Dress A, Moulton V (2006). QNet: an agglomerative method for the construction of phylogenetic networks from weighted quartets. Molecular Biology and Evolution.

[b18-ebo-4-237] Henz SR, Huson DH, Auch AF, Nieselt-Struwe K, Schuster SC (2005). Whole-genome prokaryotic phylogeny. Bioinformatics.

[b19-ebo-4-237] Huson D (1998). Splitstree- a program for analyzing and visualizing evolutionary data. Bioinformatics.

[b20-ebo-4-237] Kalmanson K (1975). Edgeconvex circuits and the traveling salesman problem. Canadian Journal of Mathematics.

[b21-ebo-4-237] Karlin S, Altschul SF (1990). Methods for assessing the statistical significance of molecular sequence features by using general scoring schemes. Proceedings National Academy of Sciences U.S.A.

[b22-ebo-4-237] Korbel JO, Snel B, Huynen MA, Bork P (2002). SHOT: a web server for the construction of genome phylogenies. Trends Genetics.

[b23-ebo-4-237] Korostensky C, Gonnet GH (2000). Using traveling salesman problem algorithms for evolutionary tree construction. Bioinformatics.

[b24-ebo-4-237] Kraskov A, Stögbauer H, Andrezejak RG, Grassberger P (2005). Hierarchical clustering using mutual information. Europhysics Letter.

[b25-ebo-4-237] Kunin V, Goldovsky L, Darzentas N, Ouzounis CA (2005a). The net of life: reconstructing the microbial phylogenetic network. Genome Research.

[b26-ebo-4-237] Kunin V, Ahren D, Goldovsky L, Janssen P, Ouzounis CA (2005b). Measuring genome conservation across taxa: divided strains and united kingdoms. Nucleic Acids Research.

[b27-ebo-4-237] Lin J, Gerstein M (2007). Whole-genome trees based on the occurence of folds and orthologs: implications for comparing genomes on different levels. Genome Research.

[b28-ebo-4-237] Makarenkov V, Leclerc B (1997). Circular orders of tree metrics, and their uses for the reconstruction and fitting of phylogenetic trees. In: Mirkin B., Morris FR., Roberts F, Rzhetsky A, (eds). Mathematical hierarchies and Biology, DIMACS Series in Discrete Mathematics and Theoretical Computer Science. Providence: Amer. Math. Soc..

[b29-ebo-4-237] Makarenkov V, Leclerc B (2000). Comparison of additive trees using circular orders. J. Computational Biol.

[b30-ebo-4-237] Makarenkov V, Kevorkov D, Legendre P (2006). Phylogenetic network construction approaches. Applied Mycology and Biotechnology, International Elsevier Series. Bioinformatics.

[b31-ebo-4-237] Mihaescu R, Levy D, Pachter L (2006). Why neighbour joining works.

[b32-ebo-4-237] Nakhleh L, Warnow T, Linder CR (2004). Reconstructing Reticulate Evolution in Species- Theory and Practice.

[b33-ebo-4-237] Pauplin Y (2000). Direct calculation of a tree length using a distance matrix. J. Mol. Biol.

[b34-ebo-4-237] Robinson W (1951). A method for chronologically ordering archaeological deposits. American Antiquity.

[b35-ebo-4-237] Saitou N, Nei M (1987). The neighbour-joining method: a new method for reconstructing phylogenetic trees. Molecular Biology and Evolution.

[b36-ebo-4-237] Snel B, Bork P, Huynen MA (1999). Genome phylogeny based on gene content. Nature Genetics.

[b37-ebo-4-237] Thuillard M (2001). Wavelets in Soft Computing.

[b38-ebo-4-237] Thuillard M (2004). Adaptive multiresolution search: how to beat brute force?. International Journal Approximate Reasoning.

[b39-ebo-4-237] Thuillard M (2007). Minimizing contradictions on circular order of phylogenic trees. Evolutionary Bioinformatics.

[b40-ebo-4-237] Timmis JN, Ayliffe MA, Huang CY, Martin W (2004). Endosymbiotic gene transfer: organelle genomes forge eukaryotic chromosomes. Nature Reviews Genetics.

[b41-ebo-4-237] Wang LS, Warnow T, Moret BME, Jansen RK, Raubeson LA (2006). Distance-based Genome Rearrangement Phylogeny. Journal of Molecular Evolution.

[b42-ebo-4-237] Wolf YI, Rogozin IB, Grishin NV, Koonin EV (2002). Genome trees and the tree of life. Trends Genetics.

[b43-ebo-4-237] Yang S, Doolittle RF, Bourne PE (2005). Phylogeny determined by protein domain content. Proceedings National. Academy of Sciences U.S.A.

[b44-ebo-4-237] Yushmanov SV (1984). Construction of a tree with p leaves from 2p–3 elements of its distance matrix (Russian). Matematicheskie Zametki.

